# Differences in the Clinical Course of COVID-19 in Patients Hospitalized in the 2023/2024 and 2024/2025 Seasons

**DOI:** 10.3390/jcm14175992

**Published:** 2025-08-25

**Authors:** Robert Flisiak, Dorota Zarębska-Michaluk, Michał Brzdęk, Marta Rorat, Krystyna Dobrowolska, Dorota Kozielewicz, Magdalena Stankiewicz, Anna Moniuszko-Malinowska, Magdalena Rogalska, Łukasz Supronowicz, Damian Piotrowski, Katarzyna Sikorska, Włodzimierz Mazur, Justyna Kowalska, Piotr Rzymski

**Affiliations:** 1 Department of Infectious Diseases and Hepatology, Medical University of Białystok, 15-540 Białystok, Poland; robert.flisiak1@gmail.com (R.F.); pmagdar@gmail.com (M.R.); l.supronowicz@wp.pl (Ł.S.); 2 Department of Infectious Diseases and Allergology, Jan Kochanowski University, 25-317 Kielce, Poland; dorota1010@tlen.pl; 3 Collegium Medicum, Jan Kochanowski University, 25-317 Kielce, Poland; krystyna.dobrowolska98@gmail.com; 4 Department of Gastroenterology, Medical University of Lodz, 92-213 Lodz, Poland; 5 Department of Social Sciences and Infectious Diseases, Medical Faculty, Wroclaw University of Science and Technology, 50-370 Wroclaw, Poland; marta.rorat@gmail.com; 6 Department of Infectious Diseases and Hepatology, Faculty of Medicine, Collegium Medicum in Bydgoszcz, Nicolaus Copernicus University, 87-100 Toruń, Poland; d.kozielewicz@wsoz.pl; 7 Department of Infectious Diseases and Hepatology, Medical University of Lublin, 20-059 Lublin, Poland; magdastankiewicz77@gmail.com; 8 Department of Infectious Diseases and Neuroinfections, Medical University of Białystok, 15-809 Białystok, Poland; anna.moniuszko-malinowska@umb.edu.pl; 9 Department of Infectious Diseases and Hepatology, Medical University of Silesia in Katowice, 40-635 Katowice, Poland; damian.piotrowski@vip.interia.pl; 10 Division of Tropical and Parasitic Diseases, Faculty of Health Sciences, Medical University of Gdańsk, 80-210 Gdańsk, Poland; ksikorska@gumed.edu.pl; 11 Clinical Department of Infectious Diseases in Chorzów, Medical University of Silesia, 41-500 Katowice, Poland; wlodek.maz@gmail.com; 12 Department of Adult’s Infectious Diseases, Medical University of Warsaw, Hospital for Infectious Diseases, 02-091 Warsaw, Poland; jdkowalska@gmail.com; 13 Department of Environmental Medicine, Poznań University of Medical Sciences, 60-806 Poznań, Poland; rzymskipiotr@ump.edu.pl

**Keywords:** SARS-CoV-2, COVID-19, Omicron, mortality, severity

## Abstract

**Background/Objectives**: The aim of this analysis of data from the multi-year nationwide SARSTer program in Poland was to compare the clinical presentation and course of COVID-19 in the last two infectious seasons. **Methods**: Clinical data from 719 consecutive patients hospitalized between April 2023 and March 2024 were compared with data from 360 patients hospitalized between 1 April 2024 and 31 March 2025. **Results**: In the 2023/2024 season, hospitalizations due to COVID-19 occurred primarily between September and January, and in the 2024/2025 season, the majority of hospitalizations occurred between July and November. In the 2024/2025 season, we documented a change in the age structure, with an increasing predominance of hospitalized patients over 70 years of age (68% vs. 60% in 2023/2024), a milder disease manifestation, reflected in a significantly lower percentage of patients with pulmonary lesions (19% vs. 24%), an improvement in the clinical course of the disease, reflected in a halving of the number of hospitalizations, a significantly higher percentage of patients with clinical improvement in subsequent weeks of hospitalization, including those discharged from the hospital within the first week (39% vs. 30%), and a significantly lower mortality rate (4.7% vs. 7.9%), especially among patients over 70 years of age (5.4% vs. 10.4%). This indicates that the trend of a milder disease course initiated by the emergence of the Omicron variant continues. **Conclusions**: In conclusion, our findings provide real-world clinical evidence of the evolution of the COVID-19 situation in the post-pandemic era.

## 1. Introduction

The Omicron (B.1.1.529) severe acute respiratory syndrome coronavirus 2 (SARS-CoV-2) lineage became globally dominant at the beginning of 2022, changing the previous image of COVID-19 as a disease that is a major threat to public health worldwide [1,2,3]. Omicron spread rapidly in many regions and, due to point mutations and recombination events, gradually evolved into numerous sublineages, which periodically dominated regionally or worldwide [1,4]. Although its subsequent subvariants were considered more contagious, they caused an increasingly mild course of the disease, expressed by a lower rate of hospitalization and intensive care unit (ICU) admissions, as well as a decreased mortality rate [5,6,7,8,9]. This phenomenon was attributed to intrinsic viral characteristics and widespread population immunity, resulting from previous infections and intensified vaccination campaigns [10]. The mass incidence of the disease in the initial period of dominance of the Omicron variant and the ability to evade vaccine and infection-induced humoral responses led to the overwhelming of healthcare systems in many countries, despite a milder clinical course of COVID-19 [11,12]. Furthermore, the emergence of immune-evasive sublineages such as BA.4/5, BQ.1, XBB, and their successors contributed to successive waves of infections, complicating predictions of the epidemic trajectory.

Previous studies assessing the clinical course and outcomes of COVID-19 have focused on the initial period of dominance of the Omicron variant, with a possible comparison to the period of dominance of the Delta and previous SARS-CoV-2 variants [5,6,7,8,9]. However, as the virus continues to circulate and evolve, real-world data across multiple seasons is needed to detect potential shifts in disease presentation, severity, or risk groups.

The present data analysis from the multi-year nationwide SARSTer program in Poland aimed to compare the clinical picture and outcomes of COVID-19 between the last two infectious seasons and to determine whether the trend towards an increasingly mild clinical course of the disease continues. Such longitudinal surveillance can also help identify signals of increased pathogenicity or immune escape that may warrant public health responses. Monitoring the clinical parameters characterizing the disease and assessing the profile of infected individuals requiring hospitalizations due to COVID-19 is important due to the continuous evolution of SARS-CoV-2 and the uncertainty about the possibility of its complete eradication [12]. The presented research results continue previous observations and can constitute a reference point for similar analyses conducted in the future. Ultimately, this ongoing monitoring helps inform evidence-based policy decisions regarding vaccination strategies, resource allocation, and the potential need for updated therapeutic guidelines.

## 2. Materials and Methods

Data were collected retrospectively, using the observational, multicenter, nationwide SARSTer database, supported by the Polish Association of Epidemiologists and Physicians of Infectious Diseases. The SARSTer database includes data from 15,130 patients, hospitalized between April 2020 and March 2025 in 30 infectious disease centers due to COVID-19, in whom the disease was diagnosed and treated under national recommendations [13,14,15]. For the purposes of this analysis, clinical data from 719 consecutive patients hospitalized between 1 April 2023 and 31 March 2024 (2023/2024 season) were compared with 360 consecutive patients hospitalized between 1 April 2024 and 31 March 2025 (2024/2025 season). According to the data available for Poland, the 2023/2024 season was dominated by the Omicron XBB.1.5 and JN.1 subvariants, while the 2024/2025 season was dominated by the JN.1, KP.3.1.1, and XEC subvariants, that as of mid-2025 are still considered by WHO as variants of interest or variants under monitoring [1,16].

The clinical course of the disease was based on patient characteristics, which included gender, age, body mass index (BMI), oxygen saturation (SpO2), clinical symptoms, lung abnormalities in imaging, comorbidities, and laboratory measures of inflammatory and coagulative activity associated with SARS-CoV-2 infection and antiviral treatment administered.

In addition, the clinical course of the disease was assessed at hospital admission and then after 7, 14, 21, and 28 days, using an ordinal scale based on the WHO recommendations, modified to an 8-point version to adapt to the specifics of the healthcare system that was previously used, the individual points of which are defined as follows: (1) not hospitalized and no activity restrictions; (2) not hospitalized, no activity restrictions, and/or not requiring oxygen supplementation at home; (3) hospitalized, not requiring oxygen supplementation, and not requiring medical care; (4) hospitalized and not requiring oxygen supplementation but requiring medical care; (5) hospitalized and requiring normal oxygen supplementation; (6) hospitalized and requiring non-invasive ventilation with high-flow oxygen equipment; (7) hospitalized for invasive mechanical ventilation or extracorporeal membrane oxygenation; and (8) death [5,7,17].

Study endpoints included length of hospital stay, need for oxygen therapy, need for mechanical ventilation, 28-day mortality, and clinical improvement defined as at least a 2-point decrease from baseline (day of hospital admission) on an ordinal scale, which were compared between seasons.

This study was conducted according to the guidelines of the Declaration of Helsinki. The SARSTer study had the approval of the Ethical Committee of the Medical University of Białystok (APK.002.303.2020). Patient consent was waived due to the retrospective design of the study.

All statistical analyses were conducted using Statistica software, version 13 (StatSoft, Tulsa, OK, USA). Categorical data were presented as counts and percentages, and comparisons between groups were performed using the chi-squared test or Fisher’s exact test, depending on the subgroup sizes. Since continuous variables did not follow a normal distribution (assessed with the Shapiro–Wilk’s test), they were described using medians and interquartile ranges. The Mann–Whitney U test was employed to compare independent groups. A *p*-value of less than 0.05 was considered statistically significant.

## 3. Results

**Figure 1:**
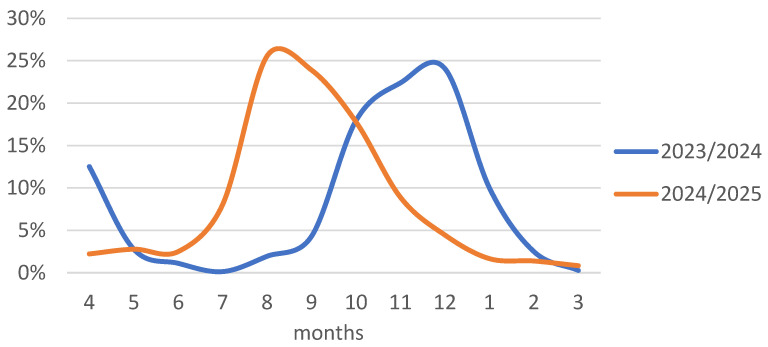
Percentage of hospitalized patients in individual months out of the total number in the 2023/2024 and 2024/2025 seasons.

The two analyzed seasons differed significantly regarding the frequency of COVID-19 hospitalizations in individual months at centers participating in the SARSTer program. While in the 2023/2024 season, hospitalizations occurred primarily between September and January, with a peak in December, in the 2024/2025 season, most hospitalizations occurred between July and November, with the highest frequency in August (Figure 1).

**Figure 2:**
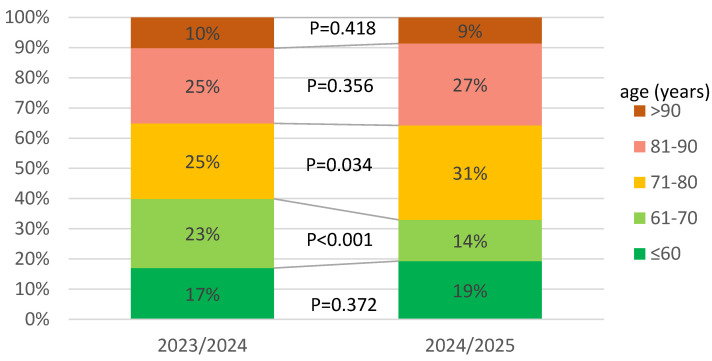
Age distribution of patients hospitalized in 2023/2024 and 2024/2025.

In infectious diseases wards participating in the SARSTer program, in the 2024/2025 season, compared to the previous season, the number of admitted patients was halved, which was accompanied by an increase in the percentage of people over 70 years of age (60% vs. 68%) (Figure 2).

In both seasons, women dominated in similar proportions, and the frequency of comorbidities was similar. The exception was significantly more frequent obesity in the last season (20% vs. 15%), which was also indicated by a significantly higher median BMI (Table 1).

There were no statistically significant differences between seasons in laboratory values of inflammatory and coagulative activity indicators associated with SARS-CoV-2 infection (Table 2).

Antiviral drugs were used with similar frequency, exceeding 40% of hospitalized patients in both seasons, although in the 2024/2025 season, the percentage of patients treated with remdesivir increased significantly, and with nirmatrelvir/ritonavir decreased; at the same time, molnupiravir therapy was discontinued. The time from symptom onset to initiation of antiviral therapy was similar in both seasons (Table 3).

Mortality in both seasons increased with age, but was significantly lower (4.7%) in the 2024/2025 season than in the preceding season (7.9%). This difference was visible in all age groups, but a statistically significant difference was found only when analyzing all subjects and in the population over 70 years of age. The analysis of the need for oxygen therapy showed an increasing trend with age, but without statistically significant differences between seasons. In turn, mechanical ventilation was used in both seasons in 2.5% and 3.1% of patients, respectively, and its frequency was similar in all age groups. The duration of hospitalization was similar in both seasons and in all age groups (Table 4).

As shown in Table 5, the frequency of the analyzed parameters assessing the outcome of the disease in people with comorbidities was similar in both seasons. Despite the mortality rate being almost half that in the 2024/2025 season (6.7% vs. 12.5%) in patients with abnormalities typical of COVID-19 shown in any imaging examination, this was not a statistically significant difference. At the same time, the length of hospitalization, the need for oxygen therapy, and mechanical ventilation were similar in both seasons (Table 5).

**Figure 3:**
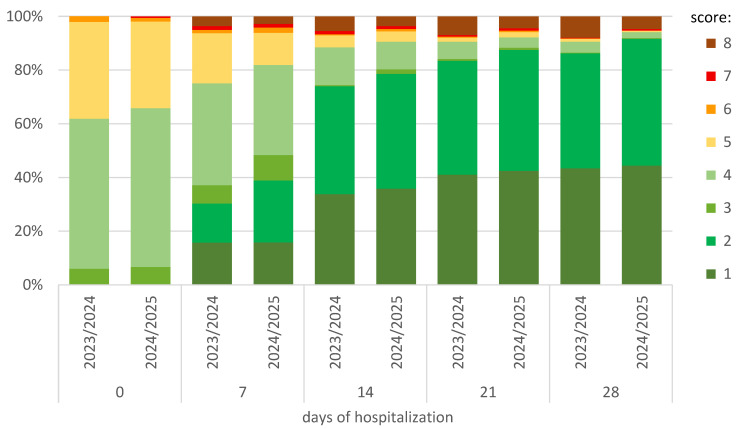
Comparison between seasons and changes at the subsequent observation timepoints of hospitalization in the ordinal-scale scoring. The score was defined as follows: (1) not hospitalized and no activity restrictions; (2) not hospitalized, no activity restrictions, and/or not requiring oxygen supplementation at home; (3) hospitalized, not requiring oxygen supplementation, and not requiring medical care; (4) hospitalized and not requiring oxygen supplementation but requiring medical care; (5) hospitalized and requiring normal oxygen supplementation; (6) hospitalized and requiring non-invasive ventilation with high-flow oxygen equipment; (7) hospitalized for invasive mechanical ventilation or extracorporeal membrane oxygenation; and (8) death.

As shown in Figure 3, in the 2024/2025 season, after the first week of hospitalization, 81.9% of patients did not require oxygen therapy (score 1–4), while in the 2023/2024 season, this percentage was 75.1% (*p* = 0.011). At the same time, 38.9% (140/360) and 30.3% (218/719; *p* = 0.005) did not require hospitalization (score 1–2), respectively. In the following weeks of observation, these differences continued to be in favor of the 2024/2025 season (Figure 3).

As shown in Table 6, the percentage of patients achieving clinical improvement at individual observation timepoints was significantly higher in those hospitalized in the 2024/2025 season.

## 4. Discussion

The emergence of the Omicron variant at the turn of 2021/2022, which was the last identified VOC of SARS-CoV-2, initiated an era of milder clinical course of COVID-19, as documented by numerous studies from real-world clinical practice [18,19]. This was particularly evident in relation to the previous period of Delta lineage dominance [5,20]. The less severe symptoms, lower mortality rate of Omicron variant infection, and increased hybrid population immunity due to vaccination and past SARS-CoV-2 infections resulted in the WHO revoking the epidemic emergency in May 2023 [21,22]. Although this does not mean that COVID-19 has been eliminated as a threat to individual health and a burden on public health, scientific and clinical interest in the disease has visibly declined, and the number of analyses assessing the clinical course of infection in the post-pandemic period is limited. Our study addresses this gap by providing a systematic comparison of two full infection seasons (2023/2024 and 2024/2025), capturing the evolving impact of SARS-CoV-2 under stable conditions of population-level immunity and in the absence of major changes to public health policy or virus classification. This focus allows us to assess the real-world burden of COVID-19 in the current healthcare environment, rather than during transitional or crisis phases. To the best of our knowledge, a comparison of the COVID-19 clinical picture and outcome over the past two infection seasons has not been performed, and our analysis is the first of its kind. By identifying patterns in disease severity, hospitalization profiles, and clinical outcomes over time, this study can support better hospital resource planning, guide expectations for patient management, and help define realistic preparedness strategies for future seasonal surges.

The current multicenter retrospective study from routine clinical practice found that there are still patients with COVID-19 who require hospital treatment. A comparison of the 2023/2024 and 2024/2025 seasons showed that the number of such individuals was halved in the latter period, suggesting that the trend of progressive attenuation of the clinical course of COVID-19 initiated by the appearance of Omicron continues regardless of its subsequent subvariants. This is consistent with observations from Catalonia documenting a significant decrease in hospitalizations due to COVID-19 in the recent 2024/2025 infection season compared to previous waves [23]. A comparison with a study analyzing the initial phase of the Omicron dominance in Poland, conducted at the same centers participating in this study, also confirms a strong downward trend in the number of hospitalizations calculated per month [7]. Among hospitalized people, elderly patients with comorbidities predominate, and in this respect, we did not find any differences between the analyzed seasons, which underscores the continued vulnerability of this population. Similar findings were also observed in the initial period of Omicron dominance [7]. Such consistent conclusions are also drawn from the numerous RWE studies conducted during the Omicron period of dominance, regardless of the subvariants [24,25]. Interestingly, women predominated among those hospitalized in both periods analyzed, with a comparable share, while it is the male gender that is a known risk factor for a more severe course of COVID-19, which translates into a risk of hospitalization [26]. This reversal of earlier patterns, manifested by the predominance of hospitalized women in the Omicron period as opposed to the Delta wave, was described in the previous analysis covering the population of Polish patients and by researchers from Slovenia [27,28]. The current study confirms the continuation of this trend.

The comparison of the 2023/2024 and 2024/2025 epidemic seasons reveals marked temporal differences in the frequency of COVID-19 hospitalizations across centers participating in the SARSTer program. While the 2023/2024 season followed a more classical autumn–winter pattern with a peak in December, the 2024/2025 season exhibited an atypical shift, with most hospitalizations occurring between July and November and peaking as early as August. This divergence underscores the increasing difficulty in predicting the timing of seasonal COVID-19 surges, especially as the virus continues to evolve and population-level immunity fluctuates due to variable vaccine uptake, waning immunity, and natural infection. Environmental factors, the emergence of new variants, and changes in human behavior, such as travel and social activities, may all contribute to these irregular patterns. Consequently, relying on historical seasonality alone is no longer sufficient to guide preparedness efforts, and continuous surveillance, flexible resource allocation, and timely public health responses remain critical to managing future COVID-19 waves.

We also showed that the clinical picture of COVID-19 has not changed significantly in the last two infection seasons. The most common clinical signs with which patients presented to the hospital were cough, fever, fatigue, and dyspnea, clinical signs described as predominant regardless of the COVID-19 wave [29,30]. A marginal and decreasing percentage of patients experienced smell and taste disorders, which were much more common in the initial period of the pandemic, and their frequency significantly decreased [7,31]. Comparing the two seasons, we also did not identify significant differences in laboratory tests. This includes the results of examinations defining the severity of COVID-19 and complications such as coagulation disorders or bacterial superinfections, such as CRP, PCT, leukocytes, IL-6, and D-dimers. The stability of these findings over time suggests a plateauing of disease severity indicators in hospitalized populations.

Noteworthy is the low median value of IL-6, a parameter whose increase in concentration during SARS-CoV-2 infection indicates the development of a cytokine storm. The development of this immune stage of COVID-19 induced by proinflammatory cytokine release was a contributor to organ damage and a predictor of fatal disease outcome [32]. During the period of Omicron dominance, regardless of the subvariant, this phenomenon is described significantly less frequently than in the earlier waves of the pandemic, when its development resulted in a deterioration of the patient’s condition, which was the direct reason for admission to the hospital [33]. During the dominance of the Omicron variant, clinical deterioration was more frequently linked to exacerbation of comorbidities rather than the infection itself. This became the immediate reason for half of the patients’ hospital admissions, as confirmed by a large Canadian study [34]. In our analysis, more than 92% of patients in both periods had comorbidities, the role of which as risk factors for a more severe course of COVID-19 has been well documented in all waves of the pandemic [35]. In line with this observation, the reason for admission was that the average saturation value at baseline remained within the normal range, and SpO_2_ below 90% was observed in only 21% of patients in the 2023/2024 season and 19% in the 2024/2025 season. Similar values with a decreasing trend over the analyzed periods were observed for abnormal CT lung scans, which correspond to the diagnosis of pneumonia in 24% and 19% of patients, respectively. The significant reduction in the proportion of patients with abnormal lung CT scans during the Omicron dominant period compared to other COVID-19 waves is well documented and is evidence supporting a milder disease course [18,19]. An analysis evaluating imaging features in hospitalized patients was also performed in the early Omicron period, comparing the dominance period of BA.1/BA.2 subvariants with BA5 [36]. The authors of this study concluded that patients infected with BA.5 had lower odds of developing radiologically confirmed pneumonia. Although the cited study did not cover the periods we analyzed, the downward trend over time aligns with the current findings. Notably, in an analysis evaluating the initial predominance of Omicron in Poland, lung involvement was described in 43–59% of patients hospitalized for COVID-19 [7]. The current data confirm a sustained and substantial decline in this parameter.

It is important to note that in our study, we did not analyze the vaccination status or prior SARS-CoV-2 infection history of patients. While these factors are known to influence the clinical course of COVID-19, including reducing the risk of pneumonia, need for oxygen supplementation, and mortality [37,38,39], their significance has evolved in the current phase of the pandemic. Under present conditions, virtually the entire adult population has already been exposed to SARS-CoV-2 antigens, either through vaccination, natural infection, or both. Consequently, the clinical and epidemiological profiles of hospitalized patients are shaped more by underlying comorbidities and age than by vaccination status alone. Though COVID-19 vaccination matters, also in the Omicron-dominated era [40,41], from the perspective of assessing the real-world burden of COVID-19 on the healthcare system, especially in a setting with near-universal antigen exposure, stratification by vaccination status may no longer yield a clinically or operationally meaningful distinction. Nevertheless, future studies aimed at evaluating individual risk factors or vaccine effectiveness may benefit from including such data.

The percentage of patients who required antiviral treatment also decreased during the early phase of Omicron’s dominance in Poland, when it was 53–63%, and this fact is further evidence of the mitigation of the course of COVID-19 over time [7]. In the present study, antiviral drugs were used with similar frequency in both analyzed periods, 40% and 44.7%, respectively, and were initiated within comparable time frames from the onset of symptoms. Due to the time-varying availability of preparations in hospital treatment, nirmatrelvir/ritonavir dominated the first period, whereas remdesivir was used more frequently in the second period. The greater use of remdesivir in the latter season coincided with a significant reduction in 28-day mortality. While our retrospective design does not allow for establishing a direct causal relationship, it is plausible that the broader administration of remdesivir, an antiviral shown to reduce progression to severe disease and mortality also in the Omicron-dominated era, particularly when used early [6,42], may have contributed to the improved outcomes observed. In contrast, nirmatrelvir/ritonavir was less commonly used in the 2024/2025 period, and molnupiravir was no longer available. These differences in antiviral strategies reflect both availability and evolving treatment preferences, and may have influenced clinical trajectories. Further comparative studies are warranted to assess the differential impact of available antiviral options in the Omicron era.

Further evidence supporting the statement about the continuous mitigation of the severity of SARS-CoV-2 infection is the reduction in the percentage of patients requiring oxygen therapy. In the initial period of Omicron dominance, it was 38–47%, and in the current analysis, oxygen therapy was utilized by 31.4% and 32.8% of patients, respectively [7]. Few patients required mechanical ventilation in both periods, but mortality decreased significantly. The decrease is evident both in relation to the initial periods of Omicron dominance (8.9–13.9%) and in the comparison of the two currently analyzed seasons. Overall, 28-day mortality in the 2023/2024 and 2024/2025 period was 7.9% and 4.7% (*p* = 0.049), respectively. This decrease was observed in every age group, but a statistically significant difference was noted in patients over 70 years. The risk of death in both seasons increased with age, being highest in patients over 90 years. This observation confirms the well-documented role of older age as a predictor of fatal outcome in COVID-19, as demonstrated in many previous analyses [43]. Although we did not document differences in the length of hospitalization, the rate of clinical improvement measured by the WHO scale was significantly higher in the 2024/2025 season at all timepoints. This is further evidence that the course of COVID-19 is becoming milder over time. We were able to demonstrate this trend through the current analysis and by comparing the data with the early days of Omicron’s dominance based on research into the same centers, which is one of the strongest points of our study. However, this study has some limitations that we have to address. These are due to the study’s real-world nature and retrospective design, which is associated with potential bias and missing data. In addition, we did not identify the specific Omicron subvariants responsible for infections during the studied periods, as we were guided by the time criterion in our analysis. Finally, we did not consider vaccination against COVID-19 because hybrid immunity resulting from vaccination and previous infection is common at this timepoint.

## 5. Conclusions

Comparing the last two COVID-19 infectious seasons, we documented an ongoing alleviation of the clinical course of the disease, expressed in a decreasing number of hospitalizations, better clinical condition of patients, faster clinical improvement, and lower mortality. These findings suggest that the trend of a milder disease course initiated with the emergence of the Omicron variant continues, likely supported by growing population-level hybrid immunity and the lower pathogenic potential of currently circulating subvariants. Despite these promising trends, COVID-19 has not been eradicated as a clinical or public health issue. Continued monitoring of viral evolution, immune escape potential, and clinical outcomes is essential, especially in vulnerable populations. Future studies should also evaluate the role of vaccination and specific subvariant circulation, which were limitations of the present analysis. Overall, our findings provide real-world clinical evidence of the evolving landscape of COVID-19 in the post-pandemic era and underscore the importance of updating clinical management strategies and resource allocation in light of changing disease dynamics.

## Figures and Tables

**Table 1:** General demographic data and clinical characteristics of patients hospitalized with COVID-19 in 2023/2024 and 2024/2025.

Parameter	2023/2024 N = 719	2024/2025 N = 360	*p*-Value
Median age, years (Q1–Q3), min–max	75 (65–84), 18–101	76 (66–84), 20–99	0.777
Sex, female/male n (%)	395/324 (55%/45%)	200/160 (56%/44%)	0.847
BMI, median (Q1–Q3)	25.8 (23.1–29.1)	26.6 (23.8–30.1)	0.030
SpO_2_ on admission, median (Q1–Q3)	95 (91–96)	95 (90–97)	0.338
SpO_2_ < 90%, n (%)	150 (20.9%)	68 (18.9%)	0.447
Abnormal CT scan, n (%)	175 (24.3%)	68 (18.9%)	0.010
Clinical symptoms, n (%):			
Cough	386 (53.7%)	188 (52.2%)	0.650
Fever	413 (57.4%)	215 (59.7%)	0.474
Fatigue	280 (33.9%)	165 (45.8%)	0.030
Dyspnea	228 (31.7%)	131 (36.4%)	0.124
Headache	73 (10.2%)	39 (10.8%)	0.730
Diarrhea	54 (7.5%)	28 (7.8%)	0.876
Vomiting	38 (5.3%)	27 (7.5%)	0.149
Nausea	25 (3.5%)	16 (4.4%)	0.433
Smell and/or taste disorders	9 (1.3%)	2 (0.6%)	0.354
Comorbidities, n (%):			
Any comorbidity	665 (92.5%)	334 (92.8%)	0.865
Hypertension	453 (63%)	233 (64.7%)	0.580
Ischemic heart disease	192 (26.7%)	89 (24.7%)	0.484
Diabetes mellitus	169 (23.5%)	87 (24.2%)	0.810
Cancer	133 (18.5%)	51 (14.2%)	0.075
Obesity (BMI > 30)	108 (15%)	72 (20%)	0.039
COPD	64 (8.9%)	26 (7.2%)	0.347
History of stroke	57 (7.9%)	34 (9.4%)	0.398

Abbreviations: BMI, Body Mass Index; CT, Computed Tomography; COPD, Chronic Obstructive Pulmonary Disease; SpO_2_, Oxygen Saturation; Q1–Q3, 1st–3rd Quartile.

**Table 2:** Laboratory parameters before treatment initiation in patients hospitalized with COVID-19.

Parameter, Median (Q1–Q3)	2023/2024	2024/2025	*p*-Value
CRP [mg/L]	43 (15.7–98)	40.6 (16–95)	0.934
IL-6 [pg/mL]	23.5 (11.7–53.8)	29.4 (14.2–60.2)	0.175
Procalcitonin [ng/mL]	0.1 (0.1–0.4)	0.1 (0.1–0.4)	0.713
WBCs [cells/μL]	6830 (4590–9370)	6870 (5400–9530)	0.156
Lymphocytes [cells/μL]	1090 (700–1600)	1110 (700–1590)	0.817
Neutrophils [cells/μL]	4760 (2800–6965)	4700 (3350–7125)	0.185
PLTs [platelets/μL]	187,000 (138,000–244,000)	189,000 (148,000–246,000)	0.217
D-dimers [ng/mL]	956 (540–1869)	998.5 (510–1891)	0.729

Abbreviations: CRP, C-reactive protein; IL-6, Interleukin-6; WBCs, White blood cells; PLTs, Platelets; Q1–Q3, 1st–3rd Quartile.

**Table 3:** Antiviral therapy used in patients hospitalized with COVID-19 in 2023/2024 and 2024/2025.

Antiviral Drug	Number (%) of Treated Patients			Time from Symptom Onset to Therapy Initiation [Days], Median (Q1–Q3)		
	2023/2024	2024/2025	*p*	2023/2024	2024/2025	*p*
Remdesivir	109 (15.2%)	138 (38.3%)	<0.001	3 (2–4)	3 (2–4)	0.234
Molnupiravir	24 (3.3%)	0	<0.001	2 (1–3)	-	NA
Nirmatrelvir/Ritonavir	155 (21.6%)	23 (6.4%)	<0.001	3 (2–4)	3 (2–4)	0.754
Total	288 (40.1%)	161 (44.7%)	0.143	3 (2–4)	5 (5–5)	0.922

Abbreviations: Q1–Q3, 1st–3rd Quartile.

**Table 4:** The outcomes of COVID-19 in hospitalized patients in the 2023/2024 and 2024/2025 seasons across the age groups.

	Season, Sample Size	Length of Hospitalization [Days], Median (Q1–Q3)	Need for Oxygen Therapy n/N (%)	Need for Mechanical Ventilation n/N (%)	Death Within 28-Day Observation n/N (%)
Total	2023/2024 N = 719	8 (6–12)	226 (31.4)	18 (2.5)	57 (7.9)
	2024/2025 N = 360	7 (5–11)	118 (32.8)	11 (3.1)	17 (4.7)
	*p*	0.104	0.655	0.597	0.049
≤60 years	2023/2024 N = 115	7 (4–10)	10 (8.7)	0	3 (2.6)
	2024/2025 N = 69	6 (4–8)	6 (8.7)	1 (1.4)	1 (1.4)
	*p*	0.701	>0.999	0.375	>0.999
>60 years	2023/2024 N = 604	8 (6–12)	216 (35.8)	18 (3.0)	54 (8.9)
	2024/2025 N = 291	8 (5–11)	112 (38.5)	10 (3.4)	16 (5.5)
	*p*	0.151	0.428	0.713	0.072
>70 years	2023/2024 N = 432	8 (6–12)	170 (39.4)	9 (2.1)	45 (10.4)
	2024/2025 N = 242	8 (6–11)	99 (40.9)	8 (3.3)	13 (5.4)
	*p*	0.121	0.692	0.443	0.025
>80 years	2023/2024 N = 252	9 (7–13)	108 (42.9)	4 (1.6)	33 (13.1)
	2024/2025 N = 130	8 (6–11)	62 (47.7)	5 (3.8)	9 (6.9)
	*p*	0.107	0.368	0.284	0.084
>90 years	2023/2024 N = 73	8 (6–10)	32 (43.8)	1 (1.4%)	14 (19.2)
	2024/2025 N = 31	8 (7–14)	17 (54.8%)	0	4 (12.9)
	*p*	0.170	0.304	>0.999	0.575

Abbreviations: Q1–Q3, 1st–3rd Quartile.

**Table 5:** Association of disease outcomes with comorbidities and lung abnormalities on any imaging modality in patients hospitalized with COVID-19 during the 2023/2024 and 2024/2025 seasons.

	Season, Sample Size	Length of Hospitalization [Days], Median (Q1–Q3)	Need for Oxygen Therapy n/N (%)	Need for Mechanical Ventilation n/N (%)	Death Within 28-Day Observation n/N (%)
Comorbidities	2023/2024, N = 665	8 (6–12)	221 (33.2)	18 (2.7)	55 (8.3)
	2024/2025, N = 334	7 (5–11)	118 (35.3)	11 (3.3)	17 (5.1)
	*p*	0.173	0.509	0.602	0.067
Lung abnormalities on imaging	2023/2024, N = 273	10 (7–13)	140 (51.9)	11 (4.0)	34 (12.5)
	2024/2025, N = 135	9 (7–14)	69 (51.1)	7 (5.2)	9 (6.7)
	*p*	0.658	0.974	0.613	0.087

Abbreviations: Q1–Q3, 1st–3rd Quartile.

**Table 6:** Clinical improvement defined as at least a 2-point decrease from the baseline on the ordinal scale at the following timepoints of hospitalization.

Season	Day 7	Day 14	Day 21	Day 28
2023/2024, N = 719	208 (28.9%)	510 (70.9)	582 (80.9)	599 (83.3)
2024/2025, N = 360	138 (38.3%)	283 (78.6%)	313 (86.9%)	327 (90.8%)
*p*	0.002	0.007	0.014	<0.001

## Data Availability

The data are available through the SARSTer database, https://sarster.pl/panel/report/report (accessed on 13 July 2025), after obtaining the coordinator’s approval.

## Abbreviations

The following abbreviations are used in this manuscript:

BMI	Body Mass Index
COVID-19	Coronavirus Disease 2019
CRP	C-Reactive Protein
CT	Computed Tomography
ECMO	Extracorporeal Membrane Oxygenation
GISAID	Global Initiative on Sharing All Influenza Data
ICU	Intensive Care Unit
IL-6	Interleukin 6
MOV	Molnupiravir
PCT	Procalcitonin
RTV	Remdesivir
RWE	Real-World Evidence
SARS-CoV-2	Severe Acute Respiratory Syndrome Coronavirus 2
SpO2	Peripheral Capillary Oxygen Saturation
VOC	Variant of Concern
WHO	World Health Organization
